# Selective Extraction of Piceatannol from *Passiflora edulis* by-Products: Application of HSPs Strategy and Inhibition of Neurodegenerative Enzymes

**DOI:** 10.3390/ijms22126248

**Published:** 2021-06-10

**Authors:** Luana Cristina dos Santos, Jose Antonio Mendiola, Andrea del Pilar Sánchez-Camargo, Gerardo Álvarez-Rivera, Juliane Viganó, Alejandro Cifuentes, Elena Ibáñez, Julian Martínez

**Affiliations:** 1 Laboratory of High Pressure in Food Engineering (LAPEA), Department of Food Engineering and Technology, School of Food Engineering, University of Campinas, R. Monteiro Lobato 80, Campinas 13083-862, Brazil; luanasantos.ea@gmail.com (L.C.d.S.); julian@unicamp.br (J.M.); 2 Laboratory of Foodomics, Institute of Food Science Research, CIAL, CSIC, Nicolás Cabrera 9, 28049 Madrid, Spain; j.mendiola@csic.es (J.A.M.); gerardo.alvarez@csic.es (G.Á.-R.); a.cifuentes@csic.es (A.C.); 3 Department of Chemistry and Food Engineering, Faculty of Engineering, University of Los Andes, Carrera 1 No. 18A-12, Bogotá 111711, Colombia; ad.sanchez@uniandes.edu.co; 4 Department of Chemical Engineering, Institute of Environmental, Chemical and Pharmaceutical Sciences, Federal University of São Paulo, R. São Nicolau 210, Diadema 09913-030, Brazil; julianevigano@gmail.com

**Keywords:** pressurized liquid extraction, Hansen solubility parameters, passion fruit by-products, piceatannol, acetylcholinesterase

## Abstract

*Passiflora edulis* by-products (PFBP) are a rich source of polyphenols, of which piceatannol has gained special attention recently. However, there are few studies involving environmentally safe methods for obtaining extracts rich in piceatannol. This work aimed to concentrate piceatannol from defatted PFBP (d-PFBP) by means of pressurized liquid extraction (PLE) and conventional extraction, using the bio-based solvents selected with the Hansen solubility parameters approach. The relative energy distance (*Ra*) between solvent and solute was: Benzyl Alcohol (BnOH) < Ethyl Acetate (EtOAc) < Ethanol (EtOH) < EtOH:H_2_O. Nonetheless, EtOH presented the best selectivity for piceatannol. Multi-cycle PLE at 110 °C was able to concentrate piceatannol 2.4 times more than conventional extraction. PLE exhibited a dependence on kinetic parameters and temperature, which could be associated with hydrogen bonding forces and the dielectric constant of the solvents. The acetylcholinesterase (AChE) and lipoxygenase (LOX) IC_50_ were 29.420 μg/mL and 27.682 μg/mL, respectively. The results reinforce the demand for processes to concentrate natural extracts from food by-products.

## 1. Introduction

Fruit by-products are strong candidates for fractionation processes aiming to recover extracts with a considerable amount of target bioactive compounds. Yellow passion fruit (*Passiflora edulis*) is one of the largest crops in tropical countries. Previous studies have demonstrated that passion fruit by-products are rich in phenolic compounds [1,2], highlighting piceatannol [3,4,5,6,7].

Piceatannol (3,3′, 4′5-Tetrahydroxystilbene, C_14_H_12_O_4_) is a natural stilbene (plant polyphenol), proven to work as an excellent antioxidant and chemoprotective compound [8]. Piceatannol is found in significant amounts in *Vitis vinifera* (wine grapes) [9], *Rhodomyrtus tomentosa* (sim fruit) [10], and in *P. edulis* seeds [11]. Despite the similarity with resveratrol’s molecule structure, piceatannol seems to exhibit improved properties, promoting health benefits that are not accounted for by resveratrol [12,13,14]. Piceatannol recovery from passion fruit seeds was reported by studies using high pressure [7] and conventional liquid extraction methods [4,6]. The mentioned works also suggested that piceatannol’s biological activity could be preserved even at high temperatures (once they applied a temperature range of 50–80 °C). Rotta et al. [6] applied hydro-alcohol extracts from passion fruit seed in dairy beverages, decreasing their lipid oxidation. In addition to its antioxidant effect, piceatannol also promoted the reduction of vascular tension [15], anti-aging effects [5], and protection against UV rays due to its inhibition effects on the production of melanin pigments [5,11]. 

More recently, piceatannol was demonstrated to have inhibitory effects on catechol-*O*-methyltransferase, an enzyme responsible for dopamine molecule breakdown [16]. Alzheimer’s disease is the main disorder of the brain that causes the deterioration of the central nervous system, affecting over 20 million people worldwide, with a growing trend as life expectation is continuously increasing [17]. Acetylcholinesterase (AChE) and lipoxygenase (LOX) play important roles in the progress of Alzheimer’s disease. On the one hand, AChE is an important enzyme in the cholinergic system, responsible for the hydrolysis of acetylcholine, a neurotransmitter distributed all over the central nervous system [18]. The main cause of Alzheimer’s is brain atrophy, which is formed by a reduction in the acetylcholine synthesis [19], and the inhibition of AChE is currently considered the main therapeutic strategy. On the other hand, the LOX pathways are associated with the lipid metabolism process, which is a key aspect of neuroinflammation. The amount of LOXs is usually regulated by the presence of the substrates arachidonic and docosahexaenoic acids, and it is believed that some of their products could worsen inflammation in brains with Alzheimer’s disease [20]. The current challenge consists of seeking novel natural compounds able to inhibit AChE and LOX and act on multiple therapeutical targets (e.g., antioxidants). In this context, this work aimed to use piceatannol as a potential inhibitor of AChE and LOX in order to complement its well-established antioxidant properties, as confirmed by oxygen radical absorbance capacity (ORAC) and ferric reducing antioxidant power (FRAP) analyses in previous studies [4,5,7,21].

To improve the solubility of a solute in a solvent, their interaction should be enhanced, and knowledge of the phase equilibria between solute (usually a solid phase) and solvent (liquid, gas, or supercritical fluid) must be acquired [22]. Therefore, one could determine the binomial pressure–temperature that would allow the proper solute solubilization in the medium to accomplish a selective extraction.

High-pressure systems are excellent alternatives for obtaining natural products, since the solvents can maintain their liquid state even above their regular boiling point, thus enhancing the compound’s solubility in the solvent [23]. In high-pressure processes, solid–liquid equilibria are usually determined experimentally or estimated by an adequate thermodynamic empirical model [22]. Hence, when the solute is expensive, it is better to find an empirical correlation suitable to the selected extraction method. Hildebrand and Scott [24] were the first to introduce the solubility parameter (δ_H_) as a tool for determining the affinity between solute and solvent. δ_H_ is based on studies of cohesion energy, which means that molecules with coincident δ_H_ values would be miscible (“like dissolves like”). However, this first empirical approach was limited to the atomic interactions based on van der Waals forces (or dispersion forces). Later, Hansen [25] (pp. 13–28) introduced a three-dimensional solubility parameter, including two other cohesion energy sources: the polar (dipole-dipole) and hydrogen-bonding forces. 

The aim of this work was to apply the Hansen solubility parameters (HSP) approach in obtaining piceatannol-enriched extracts by PLE using bio-based solvents, as well as to assess their potential ability to inhibit acetylcholinesterase and lipoxygenase enzymes.

## 2. Results and Discussion

### 2.1. Piceatannol–Solvent Affinity

The HSP estimative and calculation of relative energy distance (*Ra*) between solute and solvent molecules is detailed in Sections S0 and S1 of the supplementary material (Figure S1 and Table S1). The compounds presented in Table 1 were selected as potential solvents for the recovery of piceatannol by PLE, which are Benzyl alcohol (BnOH), Ethyl acetate (EtOAc), and ethanol (EtOH), and conventional extraction (EtOH:H_2_O, 79:21), with BnOH presenting the highest affinity to piceatannol (lower *Ra*). In order to estimate the HSPs above 25 °C, the Williams et al. [26] method was used. For liquids, Equations (S4)–(S6) (supplementary material) do not take the pressure effect into consideration as the HSPs are mainly affected by temperature if below their critical point [27]. Therefore, *Ra* for each solvent was estimated at a temperature range of 50–200 °C, as seen in Table 2 and Figure S2 (supplementary material). It is worth mentioning that to date, no work using BnOH and high-pressure extraction processes has been found in the literature. As BnOH was newly introduced as a pressurized solvent, we took its experimental isothermal density (at 25 °C) from Paknejad et al. [28] at different pressures (0.1, 1, and 10 MPa) to calculate the HSPs, as presented in Table S2 of the supplementary material. From 0.1 to 10 MPa, results show an HSP variation of only 0.50% for δ_D_, 0.21% for δ_P_, and 0.20% for δ_H_, indicating very poor influence of pressure on the HSPs.

BnOH is an aromatic alcohol often applied in cosmetics [30]. Despite its higher boiling temperature (≈205 °C) in comparison to other usual solvents, their minor residual levels that may be trapped in the product should not be a problem in food application. BnOH is considered extremely low toxic according to the European Food Safety Authority, with an acceptable daily intake (ADI) of up to 4 mg/kg body weight (bw) [31]. Thus, BnOH is classified under category A (flavoring substances that may be used in foodstuffs), according to the Committee of Experts on Flavoring Substances of the European Council [32]. The primary purpose of using BnOH in this work was to compare it with other solvents frequently used to extract bioactive compounds. Those are EtOAc, EtOH, and H_2_O, which, according to the European Directive 2009/32/CE, are part of the common extraction solvents for food applications.

### 2.2. Single-Cycle PLE: PLE Yield, Piceatannol, and Total Phenolic Content (TPC) Quantification

**Figure 1:**
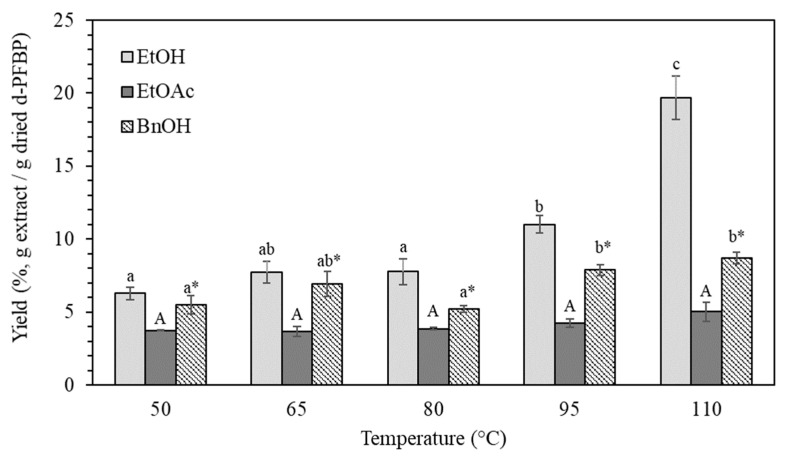
Yield of d-PFBP extract obtained by the single-cycle PLE using different solvents. Mean values that share the same lowercase without asterisk, uppercase without asterisk, or lowercase with asterisk did not show a significant difference for Ethanol (EtOH), Ethyl acetate (EtOAc), and Benzyl alcohol (BnOH), respectively, according to Tukey’s test (α = 0.05).

Supercritical fluid extraction (SFE) (35 ± 0.5 MPa, 40 ± 5 °C, 1.06 × 10^−4^ kg CO_2_/s) was essential for the removal of nonpolar content from PFBP, improving the subsequent selective phenolic extraction. This step had been already performed in a previous study that aimed to fractionate the lipid extract of PFBP [33], achieving a yield of 18.87 ± 0.12% (*w/w*). After SFE, the d-PFBP was treated using PLE. The yields for each PLE condition are shown in Figure 1.

The highest yield was achieved with EtOH at 110 °C (19.67 ± 1.47 wt %), while the lowest was for EtOAc at 65 °C (3.65 ± 0.35 wt %), where no significant variation in temperature was observed. The yield for EtOH at 65 °C (7.72 ± 0.76 wt %) was lower than that found by Viganó et al. [7] in a continuous PLE process using EtOH at 70 °C (17.518 ± 2.964 wt %). However, the same authors used a considerably high volume of solvent (since it was a continuous process), and the search for alternative solvents and less-consuming solvent processes seemed to be required in order to concentrate phenolic compounds. In this work, a trend of increasing yield with temperature is observed for EtOH. Curiously, the other solvents did not show any difference in extraction yield with temperature variation. It is important to point out that the extracts’ color intensified as the temperature was raised for all tested solvents (especially for EtOH) (Figure S3 in supplementary material). This could indicate the presence of products from sugar browning reactions at elevated temperatures, which could also have affected the yield. The conventional solid–liquid extraction achieved a yield of 7.538 ± 0.295 wt %, corroborating the results from Rotta et al. [6], who performed solid–liquid extraction in PFBP at 80 °C using EtOH:H_2_O (70:30, *v/v*) as solvent and found a yield of 8%.

The quantification of piceatannol was the starting point in outlining the following PLE extraction conditions. Table 2 presents piceatannol concentration in the extracts of d-PFBP, and the calibration curve of piceatannol (R^2^ = 0.9989) is presented in Figure S4 in the supplementary material. Despite the HSPs predictive step suggesting BnOH as the solvent with lower *Ra*, results indicated EtOH as the best solvent to concentrate piceatannol in the d-PFBP extracts. For the single-cycle PLE conditions, an increase of temperature improved piceatannol and TPC concentration in the extracts (Table 2). Such observation is corroborated by Santana et al. [4], who confirmed that piceatannol concentration is directly correlated with extraction temperature. More recently, Krambeck et al. [34] applied ultrasound-assisted extraction and reported values of around 10 μg of piceatannol/mL extract of PFBP. Similar results were obtained in this work using EtOH at 110 °C (extracts were diluted to 1 mg/mL before injection). The highest piceatannol concentration in the extracts is attributed to the conventional solid–liquid extraction, as noted in Table 2. The PLE experimental results, together with those obtained by conventional solid–liquid extraction, suggest that piceatannol has better affinity to solvents with higher polarity or their mixtures. In fact, the hydrogen bonding formation between the hydroxy groups from solute and solvent could be highly associated with the extraction of piceatannol. Viganó et al. [7] achieved 25.4 mg/g of extract using PLE with EtOH 100% at 10 MPa and 70 °C. Such result is very similar to that obtained by conventional extraction in this work. Curiously, even though the TPC content of conventional extraction was also higher, it was not proportional to the piceatannol content. It is possible that the presence of water enhanced the extraction of a wide assortment of phenolic compounds as a result of the hydrogen bonding formation.

Nevertheless, due to the differences in piceatannol concentration in conventional extraction, higher temperatures and cycles of extraction were applied, as presented in the following sections.

Some authors have performed other extraction methods to concentrate piceatannol from PFBP. Viganó et al. [7] performed PLE at 10 MPa in d-PFBP under a continuous process, using 100%, 75%, and 50% EtOH, at temperatures of 70 °C, 60 °C, and 50 °C. They reached around 6 mg/g d-PFBP using EtOH 75% at 70 °C, the same solvent-to-feed ratio (S/F) comparable with that used in this work (the cited authors did not present respective data using 100% EtOH, at a comparable S/F). Similarly, Viganó et al. [21], applying continuous PLE at the same S/F (19.7), reached around 5.5 mg/g d-PFBP using EtOH 75% at 75 °C. The differences in piceatannol concentration can be attributed to the PLE operation mode; fresh solvent flows through the extraction bed in the dynamic mode allowing for high extraction rates. Additionally, solvent composition plays an important role in extraction; according to the mentioned authors, the addition of water to ethanol (50–70% EtOH) enhanced the recovery of phenolic compounds, including piceatannol. Besides, it is well-established that different batches of raw material can present different composition.

### 2.3. Multi-Cycle PLE: Effect of Temperature and Number of Cycles

Considering the steepest ascent of higher piceatannol recovery with higher temperature observed, new extractions were performed. Table 3 presents the results for PLE at the new evaluated conditions: PLE with EtOH at 150 °C and 200 °C, and PLE in 3 cycles performed with the three tested solvents at 110 °C. A thermal stability test was carried out according to the procedure described in Section 3.3.2 to confirm that neither piceatannol nor BnOH were affected by thermal degradation at those temperatures (data shown in supplementary material, Figure S5). In order to compare the concentration of piceatannol in the extracts, results are presented in terms of mass of dried extract. The increasing temperature improved the piceatannol concentration until 150 °C, whereas at 200 °C such trend was not observed. These results suggest that PLE temperatures above 150 °C are not recommended for the concentration of phenolic compounds nor piceatannol. An increase in yield was also observed until 150 °C, but not at 200 °C, at which the degradation of some thermolabile compounds should be expected. Despite the decrease in yield, the enhanced conditions improved piceatannol concentration throughout the cycles, suggesting that a continuous process would be preferred. Besides, cycle 2 of EtOH provided the best condition, concentrating piceatannol 2.4 times more than conventional extraction. On the contrary, BnOH could not concentrate piceatannol in any of the applied conditions, while the EtOAc extract was 3.5 times more concentrated in piceatannol than BnOH. Piceatannol concentration was up to five times bigger than that obtained in single-cycle extraction, except for BnOH. Viganó et al. [7], in the best PLE condition (EtOH 50%, 10 MPa, and 70 °C), achieved a maximum piceatannol concentration of 56.5 mg/g extract. However, the present work required 10 times less solvent to achieve a very similar concentration when applying EtOH at 110 °C in cycle 2. In addition, this work was able to double the extract concentration in piceatannol when compared to the EtOH 100% condition of Viganó et al. [7]. Therefore, the application of cycles in static mode could offer some advantages over the continuous process for concentrating piceatannol. Each cycle (S/F = 19.7) lasted only 20 min, and the unneeded continuous solvent renewal makes this strategy more attractive from an environmental point of view.

The HSPs showed the smallest *Ra* for BnOH, but as shown in Table 2 and Table 3, this was not the best solvent choice to concentrate piceatannol in d-PFBP extracts within our experimental conditions. As a rule, Hansen’s model cannot be considered a determinant, but rather a preliminary solvent choice for multicomponent systems because of their complexity. Phase equilibrium between solvent and the target compounds in PLE is much more complex than in a binary system. Besides, HSPs do not consider kinetic factors. Therefore, the prediction of solvent–solute affinity may be optimistic, but the solution speed can be slower. In this sense, the results in Table 2 and Table 3 indicate a strong relationship between kinetic parameters and piceatannol extraction from d-PFBP.

It is known that the solvent power could mainly be affected by a decrease in its solubility parameter δ_H_ as temperature increases [27]. Such variation would make the solvent δ_H_ value closer to that of the solute, explaining the enhanced extraction at higher temperatures. In addition, the HSPs are basically energy parameters dependent on the dielectric constant, dipole moment, and refractive index [29]. These properties are related to the intermolecular interactions, mostly the hydrogen bonding (electron interchange), where alcohols are classified as strong proton acceptors and phenols as strong proton donors [35].

At 20 °C, the dielectric constant of H_2_O, EtOH, BnOH, and EtOAc are 80, 24.6, 13.1, and 6.0 [35], respectively, while that of the mixture EtOH:H_2_O (80:20) is 33.89 [36]. In our findings, the conventional solid–liquid extraction used mixtures of EtOH:H_2_O (79:21), thus confirming that the dielectric constant of the bio-solvents played an important role in concentrating piceatannol. Among PLE solvents, EtOH results corroborated the estimates proposed by a simple comparison among dielectric constants. However, as mentioned before, HSPs are not only predicted by a dielectric constant, but also by other important properties directly affected by temperature. In pure solvents, this property decreases as temperature increases [37]. The results in this work suggest a trend in concentrating piceatannol as extraction temperature increases, suggesting that an increase in the dielectric constant did not imply the best solvation effects on piceatannol. A possible assumption is that not only the energy of the molecule, but also its structure would interfere in intermolecular forces, changing the solvent–solute affinity.

Sánchez-Camargo et al. [38] also used HSP estimative as criteria for PLE solvent selection for obtaining fucoxanthin-enriched extracts from algae (*P. tricornutum*). The authors aimed to recover fucoxanthin, choosing d-limonene, ethanol:ethyl lactate, ethyl acetate, and CO_2_ as solvents. They found the lowest *Ra* between solute and solvent for d-limonene at 40 °C (*Ra* = 3.76). Experimentally, EtOAc and d-limonene reported the best concentration results for enriched fucoxanthin extracts. However, in terms of percent recovery, d-limonene extracts reached only 51.16% of fucoxanthin compared to conventional extraction using acetone, which presented an *Ra* of 8.64. 

**Figure 2:**
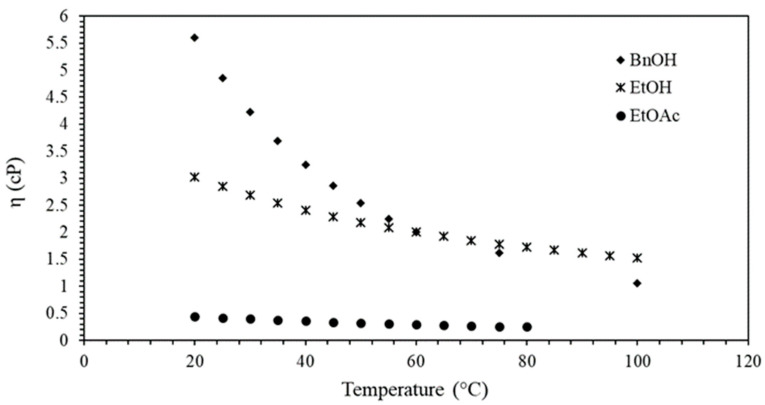
Temperature dependency of BnOH, EtOH, and EtOAc viscosities (η) at 0.1 MPa. Sources: Chemical Engineering and Material Research Information Center CHERIC [40] and Haynes [41] (pp. 6–234).

Ballesteros-Vivas et al. [39] also performed an HSP calculation to predict solvents’ suitability for mangiferin recovery from mango seed kernel. The authors concluded that ethyl lactate was the best solvent choice based on *Ra* calculation. However, a better affinity with mangiferin was found for solvent mixtures of ethanol and ethyl acetate (50:50, *v/v*). The authors also found that higher amounts of TPC were achieved at higher temperatures, corroborating the results of this work. In light of these results, a strong relationship between TPC and temperature can be inferred, whereas the solvent, rather than the temperature, could affect the recovery of a particular phenolic. Bearing in mind that the present extraction process is highly affected by temperature, solvent viscosity may play an important role in mass transfer. Figure 2 presents those variations according to thermodynamical models already reported in the literature by databank Chemical Engineering and Material Research Information Center (CHERIC) [40] and Haynes [41].

Figure 2 shows the solvent viscosities’ dependence on temperature, where EtOAc is the one with the lowest values. Despite the variations in temperature, EtOAc presented a slight decrease in viscosity, whereas BnOH and EtOH viscosities were notably reduced with temperature increase, achieving the same values at 60 °C. At 100 °C, EtOH’s viscosity proved to be higher than that of BnOH (1.52 and 1.05 cP, respectively). EtOAc yields for all tested temperatures did not present significant variations (Figure 1), not even in piceatannol concentration (Table 2), evidencing that the solubilization of the compounds presented in d-PFBP cannot be established uniquely by low viscosity. On the contrary, EtOH and BnOH showed a relationship between viscosity decrease and piceatannol concentration increase. These results indicate that solvent viscosity may not be strictly related to the extraction of piceatannol alone or phenolics, suggesting that more attention should be given to other parameters such as temperature and physical treatments.

### 2.4. Tentative Identification of Phenolic Compounds from d-PFBP by UHPLC-q-TOF-MS/MS

The phenolic profile of the *P. edulis* extract exhibiting the highest TPC concentration (PLE using EtOH at 110 °C, cycle 3) revealed a total of 25 phenolic compounds, mainly phenolic acids, flavonoids, stilbenes, carboxylic acids, and phenolic aldehydes. The tentatively annotated compounds, along with their exact mass, generated molecular formulae, calculated mass error, and MS/MS diagnostic product ions are summarized in Table 4. The proposed identification was mainly based on the comparison of experimental HRMS(/MS) data with information reported in HRMS databases (e.g., Metlin, HMDB). Acacetin and piceatannol-diglucoside were identified by comparing the experimental HR(MS/MS) data with theoretical fragmentation using CFM-ID 3.0 software. 

Citric acid, a carboxylic acid identified at the beginning of the chromatogram, was also found by Giambanelli et al. [42] in banana passion fruit pulp (*Passiflora tripartita*). The authors also performed metabolite identification using HPLC-MS/MS, reporting a citric acid retention time of 0.94 min. Many other fruits present citric acid with diverse concentrations depending on the fruit part; this compound is one of the most applied organic acids in the food industry [44]. Another phenolic acid identified in this work, dihydroxybenzoic acid, was also reported by Giambanelli et al. [42] in banana passion fruit pulp, representing 37.3% of the total bound phenolic compounds in the pulp. Caffeic and *p*-coumaric acid have also been previously found in *Passiflora cincinnata* pulp [45].

The compound at the retention time of 3.54 min was identified as catechin, showing an ion precursor [M-H]^−^ at *m/z* 289.0720. A catechin derivative (catechin hexoside) was tentatively identified at 4.1 min, and epicatechin was also found at 4.23 min with a precursor ion [M-H]^−^ at *m/z* 289.0692. Ballesteros-Vivas et al. [46] found a detailed phenolic profile of extracts obtained by PLE from *Passiflora molissima* seeds, in which catechin derivatives such as epigallocatechin, trihydroxy(iso)flavonol-(epi)catechin isomer, dihydroxyflavanol-(epi)catechin isomer, and *O*-methyl-epicatechin were tentatively identified. The retention times for such compounds were between 3.646 and 4.124 min, a range close to that found for the catechin derivatives in this work (3.54 to 4.23). The other tentatively identified flavonoids were acacetin, phloridzin, taxifolin isomers I and II, and quercetin. Some of them had not yet been reported in any other *Passiflora* species. Acacetin was identified in *Passiflora leschenaultia*, however, with amounts below the quantification limit [47]. The flavonoid quercetin was identified in extracts of *P. molissima* at 7.95 min [46], and a derivative was found in extracts of *Passiflora foetida* at 7.12 min [48]. Phloridzin was previously reported as the main flavonoid in apple seeds [49], whereas taxifolin (also called dihydroquercetin) is a common constituent in fruits and presents therapeutic potential against cardiovascular diseases [50]. 

One of the major phenolics in *P. edulis*, the stilbene piceatannol, had the precursor ion [M-H]^−^ at *m/z* 243.0647, and the same fragmentation pattern was confirmed in the Metlin experimental library. Besides, piceatannol-diglucoside was tentatively identified with the precursor ion [M-H]^−^ at *m/z* 567.1674 and the presence of fragment at *m/z* 243.0677, which is indicative of piceatannol derivative. Previous studies have evidenced piceatannol antioxidant potential in many cells [10,13,15,51]. Viganó et al. [7] emphasized the identification of piceatannol and scirpusin B as the two main phenolics in *P. edulis*. In fact, the stilbenes cis-resveratrol and scirpusin B were successfully identified in the presented work at 6.75 and 7.13 min, respectively. Resveratrol and its analogs are usually found in significant amounts in grapes [52]. Two studies using *P. edulis* reported trans-resveratrol in ethanolic seed extracts [53] and resveratrol, with the use of acetone extraction assisted by the ultrasound of *P. edulis* by-products [34]. Other stilbenes identified in this work were classified as piceatannol derivatives, and were recently found in *P. edulis* by Pan et al. [43], named passiflorinol C/D-type isomers, cyperusphenol B, passiflorinol A/B-type isomers, cyperusphenol D, and cassigarol D. The same authors elucidate the potential of such compounds as hypoglycemic agents due to the promising results in *α*-glucosidase inhibitory activities, where passiflorinol B exhibited the best results, achieving an IC_50_ = 1.7 μM. It is worth noting that this stilbene has rarely been cited in literature. Cyperusphenol B and D were isolated from *Cyperus* rhizomes by Ito et al. [54]. The same authors also applied the D isomer in leukemia (or Jurkat) cells and observed good results in suppressing their growth by the noteworthy nuclear condensation and fragmentation. Baba et al. [55] were the first authors to report the isolation of cassigarol D in *Cassia garrettiana*. Since then, few papers have identified this isomer in other plant species or evidenced their biological potential. However, some of their isomers, which include cassigarol A and cassigarol E, were found to be potential inhibitors of gastric enzymes [56] and soluble epoxy hydrolases [53]. Finally, Rasouli et al. [57] investigated how the natural polyphenols presented in plants could help with diabetes treatment. The authors found impressive results of inhibition of both *α*-amylase and *α*-glucosidase enzymes, recognizing the smaller polyphenols as the best candidates for inhibition. Some of the polyphenols tested by the mentioned authors include caffeic acid, catechin, *p*-coumaric, quercetin, and resveratrol—compounds that were also identified in the d-PFBP extracts of this work.

### 2.5. Bioactivity of d-PFBP Extracts (AChE and LOX Inhibitory Effects)

The acetylcholinesterase (AChE) and lipoxygenase (LOX) inhibition kinetics were achieved using different extract concentration ranges for the d-PFBP extracts, obtained with EtOH at 50 °C, cycles with EtOH at 110 °C, and conventional extraction. The IC_50_ values are presented in Table 5.

For AChE, the first PLE cycle was the only condition that did not achieve an IC_50_ (Section S7 in the supplementary material). From the standpoint of extraction, one possibility is that at 110 °C, the first cycle would remove a great amount of the carbohydrates from the vegetable matrix, resulting in poor AChE inhibitory capacity. Thus, once the solvent becomes more accessible to the inner parts in the subsequent cycles, the phenolics diffusion would be improved. In fact, cycles 2 and 3 achieved greater AChE inhibitory effects than other extracts. These results are possibly associated with compounds with the highest total phenolic content as well as piceatannol concentration (Table 3). Such observation is corroborated by Rege et al. [58], who stated that resveratrol is widely studied in Alzheimer’s treatment, which led us to consider the same effects in its analogues. Piceatannol standard was tested at a concentration range of 1.67–16.67 μg/mL for AChE inhibition, resulting in an IC_50_ = 10.892 ± 1.753 μg piceatannol/mL, confirming piceatannol as a potential AChE inhibitor, according to the classification stated by Santos et al. [59], against galantamine (positive control) IC_50_ = 0.89 ± 0.06 μg galantamine/mL. Such outcome reinforces the importance of developing purification processes in bioactive compounds. A recent work by Dumont et al. [60] aimed to evaluate how moderate alcohol intake (0.5 g/kg/day) associated with the administration of two stilbenes (resveratrol and piceatannol) during pregnancy and breastfeeding could affect rat fetal brains. The authors concluded that an average of 0.15 mg/kg/day of piceatannol was more beneficial than resveratrol at the same intake concentration. Despite not being fully able to protect against some types of brain damage (hypoxia-ischemia), piceatannol consumption led to a recovery of their cognitive functions after such lesions. Stilbenes were also investigated by Rivière et al. [61] on another pathway of Alzheimer’s disease—the polymerization of the amyloid β-peptide. The authors found good inhibitory effects on fibril formation associated with resveratrol, where binding on the free site of amyloid β-peptide was favorable for this phenolic molecular structure. Molecular structural investigation plays an important role in mechanisms associated with diseases, since it can reveal how molecular interactions improve the treatment, ranking the possible candidates. In this work, enrichment of a stilbene (piceatannol) in the extracts led to a decrease in AChE activity (Table 5), which is possibly related to the capability of their molecular structure to compete with the substrate (acetylcholine) in both anionic and peripheral anionic sites [59]. De Melo Filho et al. [62] reported a 96.46% AChE inhibition when applying the hexane extract of *P. foetida* at 10 mg/mL in a dimethyl sulfoxide (DMSO) solvent. However, it is possible that DMSO would not be the appropriate solvent for AChE assays since its inhibitory effects could lead results to false positive [63]. Aseervatham et al. [64] studied the aqueous extract of pulp and seeds of *Passiflora caerulea* in mice and observed good AChE inhibitory effect in the group treated with doses of the extracts at a concentration of 100 mg/kg. The authors compared the treated group to mice only administered with pilocarpine (convulsant), detecting a significant reduction in seizure severity scores. The recent results state the importance of extending the research of natural compound behavior in the cholinergic system. Besides piceatannol, other compounds present in the extract and identified in Table 4 might have exerted some AChE inhibition effects. For instance, Chohra et al. [65] have recently demonstrated that a hydro methanol extract from *Clematis cirrhosa*, rich in catechin and epicatechin, presented 78.38% of AChE inhibitory activity, which was almost 10% higher than that of another extract with smaller amounts of the same compounds. Taxifolin was also associated with therapeutic effects against Alzheimer’s disease. Saito et al. [66] investigated the cognitive function of cerebral amyloid angiopathy models in mice with and without taxifolin. The authors found very promising results as taxifolin prevented cerebrovascular deterioration in mice, also observing a decrease in Amyloid-β deposits in mice treated with this phenolic. 

Regulating LOX activity is another possible pathway associated with Alzheimer’s disease. Metabolites of this enzyme play an important role in the brain, especially linked to learning and memory processes, with 5-LOX and 12/15-LOX being the most important ones in neuroinflammatory mechanisms [20]. Among the tested conditions, extracts presented good inhibitory effects when compared to positive control quercetin (IC_50_ = 12.750 ± 0.019 μg quercetin/mL), except for the extract obtained at 50 °C. Indeed, the piceatannol standard also presented very promising results (LOX IC_50_ = 6.486 ± 0.180 μg piceatannol/mL, which is practically half of the control, quercetin). However, deeper investigation is required to better correlate a single phenolic compound with a novel therapeutical treatment of neuroinflammation. Macedo et al. [67] applied *Xylopia aethiopica* leaves extract rich in flavonoids to investigate their brain’s anti-inflammatory properties. Those extracts exhibited a high potential in decreasing LOX activity, reaching an IC_50_ of about 85 μg/mL. An investigation of nineteen different flavonoids aimed to investigate LOX inhibition [68]. Among the tested flavonoids, the authors evaluated taxifolin and epicatechin, finding only 20.9% and 37.5% LOX inhibition at a concentration of 100 μM (this work found approximately 53% LOX inhibition using piceatannol at 100 μM), which led us to assume that polyphenols and/or stilbenes are probably more related to the LOX inhibitory effects found in this work. Redrejo-Rodriguez et al. [69] also investigated how the flavonoids’ structure correlated with LOX inhibition. The authors performed a geometry optimization and concluded that the planar character of the molecules proved to be more potent inhibitors. The authors used quercetin, taxifolin, catechin, and luteolin at 100 μM, resulting in 82.7%, 22.3%, 44.1%, and 85.2% LOX inhibition, respectively.

There has been a constant investigation in the past years to replace the current treatment of Alzheimer’s disease with natural alternatives, especially using polyphenols as novel compounds in this field. However, their bioavailability needs further investigation until these compounds could be considered neuroinflammation therapeutic candidates [70]. All in all, many studies are currently putting their efforts into the discovery of natural treatments for Alzheimer’s disease, and a promising future for this application is expected.

## 3. Materials and Methods

### 3.1. Materials

Passion fruit by-products (PFBP) were kindly donated by Sítio do Belo, located in the city of Paraibuna, São Paulo, Brazil. The seed and pulp residues were defrosted at room temperature. Then, by-products were spread as thin layers on trays and submitted to dehydration in a forced convection oven at 45 ± 5 °C for approximately 48 h. The dried PFBP was milled in a conventional blender for 1 min. The milled PFBP was kept at −18 °C until the extraction process. Ethanol (EtOH), benzyl alcohol (BnOH), ethyl acetate (EtOAc) and piceatannol (purity > 98%) were bought from Cymit Quimica (Barcelona, Spain), and CO_2_ (99% purity) was bought from White Martins (Campinas, SP, Brazil). HSPiP^®^ software version 5.0 was used to determine the HSPs of the target compound (piceatannol), EtOH, EtOAc, and BnOH at 25 °C and 0.1 MPa.

### 3.2. Estimative of Hansen Solubility Parameters

The tri-dimensional solubility approach introduced by Hansen [25] was used in this work’s solubility estimative step, with piceatannol as target compound. The chosen parameter used to describe molecule affinity was the relative energy distance (*Ra*) between the target molecule and the solvent (or their mixtures). A step-by-step calculation for solute and solvent is presented in the Section S0 of the supplementary material.

### 3.3. Extraction Procedures

#### 3.3.1. Supercritical Fluid Extraction: Defatting Step

Supercritical fluid extraction (SFE) was performed as described by Viganó et al. [71] with some modification. About 50 g of dried and milled seeds were packed into a 100 mL stainless steel vessel. The SFE condition was defined based on the authors’ maximum extract yield: 35 ± 0.5 MPa, 40 ± 5 °C, and a solvent flow rate of 1.06 × 10^−4^ kg CO_2_/s. The static time was 30 min, and a previous kinetic study allowed us to establish an S/F ratio of 46 kg CO_2_/kg dried PFBP [33]. The lipid extract was stored for other purposes, while the defatted PFBP (d-PFBP) was kept at −18 °C until further steps. 

#### 3.3.2. Pressurized Liquid Extraction (PLE)

PLE was carried out in an ASE 200 (Dionex, Sunnyvale, CA, USA). Approximately 1 g of raw material was compacted in an 11 mL stainless steel cell filled with two equal parts of approximately 1 g of sea sand. PLE was performed at 1500 psi (10.3 MPa) at five different temperatures (50, 65, 80, 95, and 110 °C), using the solvents determined in the predictive step (Section 3.2), namely EtOH, EtOAc, and BnOH. After static time (20 min), extraction was followed by 60 s of flushing with nitrogen. Approximately 25 mL of solvent was used, resulting in an S/F (solvent-to-feed mass ratio) of 19.7, 22.5, and 26.1 for EtOH, EtOAc, and BnOH, respectively. The extracts were collected in a vial glass covered with aluminum foil to prevent from light oxidation. Total extraction yield (100 × mass of extract/mass of dried feed) of EtOH and EtOAc was achieved by residual solvent evaporation under constant N_2_ flow at 25 °C, while BnOH had the total volume extraction measured, and aliquots of 1 mL were taken and evaporated under an N_2_ stream in a thermostatic Turbovap LV evaporation station (Biotage, Uppsala, Sweden) at 90 °C. Later, extracts were resuspended in EtOH at a concentration of 10 mg/mL (except BnOH extracts kept in the same solvent at their particular concentrations) and kept under −20 °C.

The solvent that best concentrated piceatannol was further enhanced following Steepest Ascent Method for Optimization, at two higher temperatures: 150 and 200 °C. Later, three PLE cycles were performed in d-PFBP for each solvent, without replacing the raw material in the system. 

Due to the lack of previously published information on the thermal stability of piceatannol, and considering the possibility of oxidation of BnOH in the experimental range of temperatures used, thermal stability tests were carried out as follows: a stock solution of 100 µg/mL of piceatannol in BnOH was prepared and divided into different encapsulated vials sealed with nitrogen atmosphere and stored at different temperatures (room temperature, 80 °C, 150 °C, and 200 °C) for 30 min; later on, each of them was injected in HPLC-DAD using the method described in Section 3.4.1. 

#### 3.3.3. Conventional Solid–Liquid Extraction

The conventional method selected for comparison with the PLE is described by Lai et al. [72]. Briefly, approximately 5 g of d-PFBP and 30 mL of EtOH:H_2_O (79:21) were inserted into falcon tubes and shaken in a thermo-mixer Eppendorf (Wesseling, Germany) at 85 °C for 80 min at 250 rpm. The supernatant was collected and 5× *g*, centrifugated and the extracts were kept under freezing (−20 °C) until further analysis. This procedure was carried out in duplicate.

### 3.4. Extract Characterization

#### 3.4.1. High-Performance Liquid Chromatography with a Diode-Array Detector (HPLC-DAD): Piceatannol Quantification

Quantification of piceatannol was performed following the method described by Lai et al. [10], with some modification. The analyses were performed in an HPLC-DAD Agilent 1100 series (Santa Clara, CA, USA), using a C18 Agilent Poroshell 120 column (100 mm × 4.6 mm i.d., 2.7 μM particle size; Santa Clara, CA, USA). The mobile phases were water with 0.1% formic acid (solvent A) and acetonitrile with 0.1% formic acid (solvent B). The compounds were separated through the column with temperature set at 35 °C, and according to the solvent gradient: 10–20% B (0–2 min); 20–35% B (2–15 min); 35–60% B (15–21 min); 60–10% B (21–22 min), and 10% B (22–25 min). The flow rate was 0.75 mL/min, while the volume of injection was 20 μL. Absorbance was recorded at 324 nm, and spectra from 240 to 770 nm were recorded using a diode-array detector. For injection, all the extracts were diluted to the concentration of 1 mg/mL in EtOH and filtered using a nylon syringe filter with a pore size of 0.22 μM. The calibration curve of piceatannol was achieved using the same method parameters in a concentration range of 1–200 ppm in EtOH. 

#### 3.4.2. Total Phenolic Content (TPC) 

TPC was estimated according to the Folin-Ciocalteu assay [73]. Briefly, a 10 µL aliquot of extract solution (concentration ranging from 1 to 10 mg/mL) and 600 µL of water milli-Q were mixed, to which 50 µL undiluted Folin-Ciocalteau reagent (Merck, Darmstadt, Germany) was subsequently added. After 1 min, 150 µL of 20% (*w/v*) Na_2_CO_3_ was added, and the volume was increased to 1 mL, with water. After 2 h incubation, 300 µL of each reaction mixture was transferred to a 96-well microplate. The absorbance was measured at 760 nm in a microplate spectrophotometer reader Synergy HT (BioTek Instruments, Winooski, VT, USA). The calibration curve with gallic acid solutions in water was used in a concentration range of 0.031–2 mg/mL GA. The results were presented as the average of a triplicate analysis expressed as mg gallic acid equivalents (GAE)/g extract.

#### 3.4.3. Phenolics Profile of d-PFBP Extract Using UHPLC-q-TOF-MS/MS 

An ultra-high performance liquid chromatography Agilent 1290 UHPLC system, coupled to a quadrupole time-of-flight mass spectrometer (q-TOF-MS) and equipped with an electrospray ionization (ESI) source operated in negative mode (ESI-) was used for the phenolic profiling of PFBP extracts. The separation was set at 30 °C in a Zorbax Eclipse Plus C18 column (100 mm × 2.1 mm, 1.8 μM particle size) (Agilent Technologies, Santa Clara, CA, USA). Mobile phases were water (A) and Acetonitrile (B), containing 0.01% of formic acid. Flow rate and sample injection volume was 0.5 mL/min and 5 μL, respectively. Elution gradient was as follows: 0 min–0% B; 7 min–30% B; 9 min–80% B; 11 min–100% B; 13 min–100% B; 14 min–0% B. The mass spectrometer was operated in HRMS (high-resolution MS) and HRMS/MS modes for the structural analysis of phenolic compounds. MS parameters were the following: capillary voltage, 4000 V; nebulizer pressure, 40 psi; drying gas flow rate, 10 L/min; gas temperature, 350 °C; skimmer voltage, 45 V; fragmentor voltage, 110 V. The MS and Auto MS/MS modes were set to acquire *m/z* values at the ranges of 50–1100 and 50–800, respectively, at a scan rate of 5 spectra/s. 

For post-acquisition data processing, Agilent Mass Hunter Qualitative Analysis software (version B.07.00, Agilent, Santa Clara, CA, USA) was used, applying data mining strategies reported by Ballesteros-Vivas et al. [39]. A preliminary list of compounds was created based on previously reported studies. Tentative identification was proposed comparing MS and MS/MS data with information reported in MS databases (e.g., Metlin, HMDB) and in literature. Otherwise, predictive fragmentation through competitive fragmentation modeling by CFM-ID 3.0 software (Wishart Lab, Edmonton, AB, Canada) was used. When available, commercial standards were used for identity confirmation.

### 3.5. Bioactivities of d-PFBP Extracts

#### 3.5.1. Acetylcholinesterase (AChE) Inhibition

The AChE inhibitory effects were measured following the methodology described by Ellman et al. [74], with the adaptations proposed by Yu et al. [75]. Extract samples (0.25–1.5 mg/mL) or positive control galantamine (0.0125 mg/mL) (Sigma-Aldrich, Madrid, Spain) were dissolved in EtOH:H_2_O (1:1, *v/v*) solution, in that order. The substrate acetylthiocholine iodide (ACth) (Sigma-Aldrich, Madrid, Spain) was diluted in water milli-Q. Reagent 4-fluoro-7-sulfanoylbenzofurazan (ABD-F) (TCI Chemicals, Tokyo, Japan) was diluted in buffer Tris-HCl 150 mM (pH = 8); stock enzyme solution (AChE) type VI-S from Electrophorus electricus (Sigma-Aldrich, Madrid, Spain) was prepared in Tris-HCl 150 mM (pH = 8) with 0.1% of BSA (bovine serum albumin) (Sigma-Aldrich, Madrid, Spain). Firstly, ACth concentrated at 3.2 mM was used to determine the enzymatic velocity constant (or K_m_). ACth concentrations ranged between 0.1067 and 1.0667 mM/μL. Sequentially, 50 μL of EtOH:H_2_O (1:1, *v/v*), 25 μL of ABD-F at 0.125 mM, 100 μL of buffer (pH = 8), and 25 μL of the enzyme AchE at 0.8 U/mL were added in a black 96-well-microplate in triplicate, which was read in a microplate reader SynergyHTX (BioTek Instruments, Winooski, VT, USA) every 10 s for 15 min. The adjusted spectrophotometric parameters given were: temperature = 37 °C; wavelength excitation = 389 ± 20 nm, and emission = 513 ± 20 nm. The K_m_ constant was determined by the substrate concentration needed to achieve half of the maximum enzyme reaction velocity. The AChE inhibition assays were assembled using the same reaction principle, respecting the following order: (I) Extracts concentrated in a range 50–500 μg/mL; (II) 100 μL of buffer (pH = 8); (III) 25 μL of AChE solution at 0.8 U/mL; (IV) 25 μL of ABD-F solution; and (V) 50 μL of ACth solution at the calculated K_m_. An incubation period of 10 min in the absence of light was done after step III. The microplate reading parameters were the same as those described previously to determine K_m_. The same procedure was performed, substituting the extract for galantamine (0.4–4 μg/mL) for comparison. Values were obtained in triplicate, and the calibration curves were built to obtain the AChE inhibition percentage, which was calculated according to Equation (1). Results were expressed as IC_50_ (%) (i.e., extract concentration required to inhibit the activity of AChE in 50%).

(1) $$\%\mathrm{Inhibition}=100\;\times \;({\bar{V}}_{blank}\;-\;{\bar{V}}_{i})/{\bar{V}}_{blank}$$

${\bar{V}}_{i}$ is the calculated mean of the enzymatic velocity at *i* extract concentration, and ${\bar{V}}_{blank}$ is the mean velocity of the enzyme when no extract was added.

#### 3.5.2. Lipoxygenase (LOX) Inhibition

The LOX assay followed a similar procedure presented for the AChE assay, with some modification. The buffer used was Tris-HCl 150 mM with pH = 9, enzyme lipoxygenase (TCI Chemicals, Tokyo, Japan) was solubilized in a buffer (0.208 U/mL). The substrate was linoleic acid (Sigma-Aldrich, Madrid, Spain) stock solution 7 mM in EtOH:H_2_O (1:4) (before Km calculation) and the reagent was fluorescein 1 μM. Extracts were dissolved in EtOH:H_2_O (1:4) until 0.25–1.5 mg/mL. After adding the extract solution into the specified concentrations (7.14–428.57 μg/mL, depending on the sample), 75 μL of fluorescein, 100 μL of the substrate, and 75 μL of the enzyme were added in a black 96-well-microplate and read in a microplate reader SynergyHTX (BioTek Instruments, Winooski, VT, USA) every 10 s for 15 min. The adjusted spectrophotometric parameters given were: temperature = 27 °C, wavelength excitation = 485 ± 20 nm, and emission = 530 ± 20 nm. In order to compare inhibition values with the positive control, the same procedure was performed replacing the extract by quercetin (Sigma-Aldrich, Madrid, Spain) at 0.85 mg/mL (25–250 μg/mL). Values were obtained in triplicate, and the calibration curves were built to obtain LOX inhibition percentage, which was calculated according to Equation (1). Results were expressed as IC_50_ (%) (i.e., extract concentration required to inhibit LOX activity at 50%).

### 3.6. Statistical Analysis 

Analysis of variance (ANOVA) and Tukey’s test (at 95% confidence level) were performed using Statistica 10 (StatSoft, Tulsa, OK, USA), to verify that the solvents and temperatures tested had a statistically significant effect on the yield, piceatannol concentration, and TPC responses. 

## 4. Conclusions

In the search for green solvents for the extraction of piceatannol from d-PFBP by their Hansen Solubility parameters, EtOH, EtOAc, and BnOH were chosen for PLE. BnOH presented the smaller *Ra*, but experimental results showed that this was not the best solvent to concentrate piceatannol from d-PFBP within the tested temperature range. EtOAc was not selective for piceatannol recovery under applied conditions. On that account, EtOH was the preferred solvent over the others, using PLE at 110 °C. The results suggested that piceatannol extraction from d-PFBP was more dependent on temperature than on solvent affinity (*Ra*), which could be directly associated with the hydrogen bonding forces and the dielectric constant of the bio-solvents. PLE cycles were able to concentrate the extract in total phenolics and piceatannol, i.e., cycles 2 and 3 presented higher concentrations of such compounds as compared to the first cycle, indicating a strong relationship between kinetic parameters and piceatannol extraction from d-PFBP. Moreover, extracts showed important bioactivities. AChE and LOX inhibition presented the best results for extracts from PLE using EtOH at 110 °C, at both cycles 2 and 3, as no significant difference was reported. Therefore, the results suggested that PLE using EtOH at 110 °C and two extraction cycles could be efficient in obtaining a piceatannol-concentrated extract (55.5 ± 6.9 mg/g), although further in vivo investigation associated with bioavailability and toxicity is desired. Finally, this work showed d-PFBP as a promising source of bioactive compounds against neurodegenerative diseases and reinforced the demand for processes to concentrate natural extracts.

## Figures and Tables

**Table 1:** HSP * values, temperature of the boiling point (T_b_), and relative energy distance (*Ra*) between target compound and solvents (25 °C and 0.1 Mpa). (Calculated using HSPiP^®^).

	Molecular Structure	Molecular Formula	T_b_ (°C) (at 0.1 Mpa)	δ_D_ (Mpa^1/2^)	δ_P_ (Mpa^1/2^)	δ_H_ (Mpa^1/2^)	δ_T_ (Mpa^1/2^)	*Ra* (Mpa^1/2^)
Piceatannol (target)	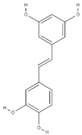	C_14_H_12_O_4_	-	21.3	7.0	10.4	24.7	0
BnOH	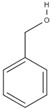	C_7_H_8_O	205	18.4	6.3	13.7	23.8	6.7
EtOAc	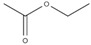	C_4_H_8_O	77	15.8	5.3	7.2	18.2	11.6
EtOH	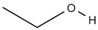	C_2_H_5_OH	78	15.8	8.8	19.4	26.5	14.3
EtOH:H_2_O (79:21)	-	-		15.7	10.3	24.2	31.0	18.0

* Hansen Solubility Handbook [29] (pp. 173–190).

**Table 2:** Piceatannol content and TPC in d-PFBP extracts obtained by PLE using different solvents and conventional solid–liquid extraction.

Extraction	Solvent	T (°C)	*Ra* Value	Piceatannol (mg/g of Dried d-PFBP)	Piceatannol (mg/g of Dried Extract)	TPC (mg GAE/g of Dried Extract)
PLE (10 Mpa)	EtOH	50	14.53	n.q.	n.q.	34.00 ± 0.08 ^d^
		65	14.72	n.q.	n.q.	n.d.
		80	14.96	0.23 ± 0.03 ^d^	3 ± 0 ^c^	n.d.
		95	15.25	0.49 ± 0.03 ^c^	4.48 ± 0.05 ^c^	n.d.
		110	15.59	1.43 ± 0.03 ^b^	11.0 ± 1.8 ^b^	91.4 ± 11.4 ^b^
	EtOAc	50	12.36	n.q.	n.q.	11.77 ± 0.25 ^d^
		65	12.84	n.q.	n.q.	n.d.
		80	13.33	n.q.	n.q.	n.d.
		95	13.83	0.128 ± 0.002 ^d^	3.130 ± 0.295 ^c^	n.d.
		110	14.36	0.268 ± 0.007 ^d^	6.24 ± 0.36 ^c^	65.1 ± 8.5 ^c^
	BnOH	50	6.87	n.q.	n.q.	24.68 ± 2.55 ^d^
		65	6.99	n.q.	n.q.	n.d.
		80	7.13	n.q.	n.q.	n.d.
		95	7.27	0.27 ± 0.04 ^d^	3.4 ± 0.7 ^c^	n.d.
		110	7.42	0.48 ± 0.04 ^c^	5.6 ± 0.2 ^c^	65.05 ± 8.03 ^c^
Solid–liquid	EtOH:H_2_O (79:21)	85	17.6	1.81 ± 0.07 ^a^	23.4 ± 1.5 ^a^	269.6 ± 4.4 ^a^

Equal letters in the same column indicate no significant difference between the mean values, according to Tukey’s test (α = 0.05). n.q.: not quantified (peak area < 1 ppm calibration curve correspondent). n.d.: not determined.

**Table 3:** d-PFBP extracts characterization in multi-cycle PLE and higher temperatures using different bio-solvents.

Extraction	Solvent	T (°C)	*Ra* Value	Cycle	Yield (%, g/g of Dried d-PFBP)	Piceatannol (mg/g of Dried Extract)	TPC (mg GAE/g of Dried Extract)
PLE (10 MPa)	EtOH	110	15.59	1	19.7 ± 1.5 ^ab^	11.0 ± 1.9 ^d^	91.4 ± 11.4 ^g^
				2	3.17 ± 0.06 ^ef^	55.5 ± 6.9 ^a^	419.18 ± 10.15 ^b^
				3	1.80 ± 0.08 ^f^	56.5 ± 4.6 ^a^	490.1 ± 13.3 ^a^
		150	16.77	1	23 ± 1 ^a^	12.26 ± 0.16 ^d^	206 ± 5 ^de^
		200	18.71	1	19.1 ± 2.2 ^b^	10.7 ± 0.8 ^d^	177 ± 7 ^ef^
	EtOAc	110	14.36	1	5.0 ± 0.7 ^de^	6.2 ± 0.4 ^d^	65 ± 8 ^g^
				2	0.980 ± 0.015 ^f^	23.8 ± 0.6 ^bc^	158 ± 12 ^f^
				3	0.51 ± 0.05 ^f^	32 ± 2 ^b^	178 ± 20 ^ef^
	BnOH	110	7.42	1	8.7 ± 0.4 ^c^	5.6 ± 0.2 ^d^	65 ± 8 ^g^
				2	2.8 ± 0.1 ^ef^	6.09 ± 0.07 ^d^	176 ± 34 ^ef^
				3	1.8 ± 0.2 ^ef^	9.2 ± 0.7 ^d^	225 ± 43 ^cd^
Solid-Liquid	EtOH:H_2_O (79:21)	85	17.6	-	7.5 ± 0.3 ^cd^	23.4 ± 1.5 ^c^	270 ± 4 ^c^

Equal letters in the same column indicate no significant difference between the mean values, according to Tukey’s test (α = 0.05). TPC: Total phenolic content.

**Table 4:** Tentatively identified compounds in d-PFBP extract (PLE with EtOH at 110 °C, cycle 3).

Tentative Identification	RT (Min)	Molecular Formula	Calculated [M-H]^−^ (*m/z*)	Experimental [M-H]^−^ (*m/z*)	Error (ppm)	MS/MS Product Ions (*m/z*)	Reference ^a^
Citric acid isomer I	0.60	C_6_H_8_O_8_	191.0197	191.0199	1.0	85.0312, 57.0367, 87.0111, 111.0102	[42]
Citric acid isomer II	0.92	C_6_H_8_O_8_	191.0197	191.0193	−2.1	87.0112, 111.0118, 85.0324, 57.0371	[42]
Acacetin	2.03	C_16_H_12_O_5_	283.0612	283.0615	1.1	151.0278, 283.0685	[CFM-ID]
Dihydroxybenzoic acid	2.41	C_7_H_6_O_4_	153.0193	153.0194	0.7	109.0318, 108.0251, 91.0214, 65.0055	[42]
4-Hydroxybenzoic acid	3.11	C_7_H_6_O_3_	137.0244	137.0243	−0.7	93.035, 65.0418	[Std]
Catechin	3.54	C_15_H_14_O_6_	289.0718	289.0720	0.7	123.0467, 203.0718, 245.0843,	[Std]
Caffeic acid	3.81	C_9_H_8_O_4_	179.0350	179.0357	3.9	133.0322, 79.0585, 89.0429	[Std]
Taxifolin isomer I	3.84	C_15_H_12_O_7_	303.0510	303.0505	−1.6	175.0368, 285.0424	[Metlin]
Catechin hexoside	4.10	C_21_H_24_O_11_	451.1246	121.0300	4.1	289.0741	[42]
Piceatannol diglucoside	4.15	C_26_H_32_O_14_	567.1719	451.1228	−4.0	405.1208, 243.0677, 406.1224	[CFM-ID]
Epicatechin	4.23	C_15_H_14_O_6_	289.0718	567.1699	−3.5	123.0466, 203.0718, 245.0843	[Metlin]
*p*-Coumaric acid	4.69	C_9_H_8_O_3_	163.0401	163.0398	−1.8	119.0364, 163.0201, 147.0334	[Std]
Phloridzin	4.70	C_21_H_24_O_10_	435.1297	435.1284	−3.0	273.0807, 123.0475	[Metlin]
Passiflorinol C/D-type	4.90	C_29_H_24_O_10_	531.1296	531.1289	−1.5	362.0812, 265.0533	[43]
Passiflorinol C/D-type isomer	5.38	C_29_H_24_O_10_	531.1296	531.1288	−1.6	283.0642, 165.0226	[43]
Taxifolin isomer II	5.47	C_15_H_12_O_7_	303.0510	303.0502	−2.6	125.027, 175.0411, 285.0438	[Metlin]
Cyperusphenol B	5.56	C_42_H_32_O_12_	727.1821	727.1795	−3.6	495.1126, 373.0740, 265.0542	[43]
Piceatannol	5.72	C_14_H_12_O_4_	243.0663	243.0667	1.6	243.0696, 159.0476, 201.0588	[Std]
Passiflorinol A/B-type	6.09	C_42_H_32_O_12_	727.1821	727.1803	−2.5	617.1471, 361.0748, 243.0681	[43]
Tetrahydroxy(iso)flavanone	6.30	C_15_H_12_O_6_	287.0562	287.0567	1.7	259.0632, 125.0272	[Metlin]
cis-Resveratrol	6.75	C_14_H_12_O_3_	227.0714	227.0718	1.8	143.0521, 227.0735, 185.0654	[Metlin]
Scirpusin B	7.13	C_28_H_22_O_8_	485.1242	485.1228	−2.9	485.1289, 375.0916, 486.1316	[7]
Passiflorinol A/B-type isomer	7.19	C_42_H_32_O_12_	727.1821	727.1805	−2.2	618.1516, 483.1165, 373.0758, 243.0709	[43]
Cyperusphenol D	7.36	C_42_H_30_O_12_	725.1665	725.1640	−3.4	617.1509, 481.0925, 373.0747, 243.0685	[43]
Quercetin	7.56	C_15_H_10_O_7_	301.0354	301.0352	−0.7	151.0059, 179.0006, 301.0385	[Std]
Passiflorinol A/B-type isomer	7.92	C_42_H_32_O_12_	727.1821	727.1800	−2.9	617.1516, 495.1102, 373.0747, 241.0555	[43]
Cassigarol D isomer I	8.10	C_28_H_20_O_8_	483.1085	483.1062	−4.9	243.0695, 201.0571	[43]
Cassigarol D isomer II	8.88	C_28_H_20_O_8_	483.1085	483.1072	−2.8	295.0637, 241.0519	[43]

^a^ Identification based on compounds reported in literature [reference in brackets]; structural similarity based on MS-Databases [Metlin] or theoretical fragmentation [CFM-ID]; reference standard [Std].

**Table 5:** AChE and LOX IC_50_ values for d-PFBP extracts.

Extraction	Solvent	T (°C)	Cycle	AChE IC_50_ (μg/mL)	LOX IC_50_ (μg/mL)
PLE (10 MPa)		50	1	395.63 ± 9.774 ^a^	211.689 ± 12.279 ^a^
	EtOH	110	1	n.d.	40.478 ± 0.597 ^b^
			2	43.297 ± 3.249 ^bc^	32.035 ± 1.355 ^b^
			3	29.420 ± 1.615 ^c^	27.682 ± 2.477 ^b^
Solid-Liquid	EtOH:H_2_O (79:21)	85	-	58.87 ± 0.05 ^b^	29.720 ± 2.627 ^b^

n.d.: not determined (maximum level of inhibition below 50%). Equal letters in the same column indicate no significant difference between the mean values according to Tukey’s test (α = 0.05).

## Data Availability

Not applicable.

## Supplementary Materials

The supplementary materials are available online at https://www.mdpi.com/article/10.3390/ijms22126248/s1.
