# Supplementary material for: Selective Extraction of Piceatannol from Passiflora edulis by-Products: Application of HSPs Strategy and Inhibition of Neurodegenerative Enzymes
Source: Int J Mol Sci. 2021 Jun 10;22(12):6248. doi: 10.3390/ijms22126248 (PMC8230382; doi:10.3390/ijms22126248)
Supplement: Supplementary file 1 [file ijms-22-06248-s001.zip › ijms-1205465-supplementary.pdf]

Supplementary material

S0. Step-by-step equations for HSPs calculation

The relative energy distance (*Ra*) between the target molecule and the solvent (or their mixtures) was calculated according to Equation S1.

$$R_a = \sqrt{4\left(\delta_{Di} - \delta_{Dj}\right)^2 + \left(\delta_{Pi} - \delta_{Pj}\right)^2 + \left(\delta_{Hi} - \delta_{Hj}\right)^2} \qquad \text{(S1)}$$

where, δ_D is the energy density from dispersion bonds between molecules, δ_P is the energy from dipolar intermolecular force between molecules, and δ_H is the energy from hydrogen bonds between molecules, i = solute and, j = solvent.

HSPiP® software (Version 5.0, UK) was used to test the affinity of piceatannol with more than 2000 compounds from the EAFUS (Everything added to food in the United States) data base list, by means of Yamamoto – Molecular Break (Y-MB) method included in the software. Y-MB method uses the simplified molecular input line syntax (SMILES) of each compound for the HSP estimations. The software's temperature and pressure limitations were overcome estimating the HSPs and the Ra value, using some empirical models available in literature as described as follows.

a) HSPs: Target compound

In order to evaluate the temperature effects in the HSPs of piceatannol (considering that pressure effects are neglected in solids), the methodology presented by Jayasri and Yaseen was used (Equation S2).

$$\delta_2 = \delta_1 \left(\frac{1 - T_{r2}}{1 - T_{r1}}\right)^{0.34} \qquad \text{(S2)}$$

where, *Tr* refers to the reduced temperature at 25 °C (1) at a given sub temperature (2). For the calculation of the critical temperature of piceatannol (Tc), Marrero and Gani group contribution method was used, as described in Equation S3.

$$exp\left(\frac{T_c}{T_c^0}\right) = \sum_i N_i C_i + w \sum_j M_j D_j + z \sum_k O_k E_k \qquad \text{(S3)}$$

where, T_c^0 is the universal constant = 231.239 K, N_i, M_j, and O_k are the number of groups from first, second, and third-order, respectively C_i, D_j, and E_k are the contribution value of each group and, w and z are zero or the unit, depending on the groups' presence in the molecule.

Once the critical temperature was calculated, as well as the HSPs at 25 °C was obtained in the HSPiP® software, it was possible to calculate all three HSPs (δ_D, δ_P and, δ_H) for piceatannol from 25 to 150 °C using Equation S2.

b) HSPs: Solvents

Three bio-solvents (BnOH, EtOAc, and EtOH) and one mixture of EtOH and H_2O (79:21, v/v) were selected as of the most suitable solvents for solubilization of piceatannol, according to their *Ra* values (between 6.7 to 18.0) for further calculations. Their HSPs at different temperatures and pressures was calculated using the methodology of Williams, Rubin, and Edwards [26]. These authors explored high pressure and temperature conditions, including the supercritical

region, verifying data consistency through the total solubility parameter comparison. The integrated results are shown in Equations S4(a-c).

$$\frac{\delta_{Dref}}{\delta_D} = \left(\frac{V_{ref}}{V}\right)^{-1.25} \quad \text{(S4-a)}$$

$$\frac{\delta_{Pref}}{\delta_P} = \left(\frac{V_{ref}}{V}\right)^{-0.5} \quad \text{(S4-b)}$$

$$\frac{\delta_{Href}}{\delta_H} = exp\left[-1.32 \times 10^{-3}\left(T_{ref} - T\right) - \ln\left(\frac{V_{ref}}{V}\right)^{0.5}\right] \quad \text{(S4-c)}$$

where, T_{ref} is the reference temperature (in K) in which the parameter was estimated and, V_{ref} is the molar volume at the corresponding reference temperature.

Equations S4(a-c) require previous estimative of HSPs at a reference temperature. This work's reference was the temperature set for HSPs calculation (25 °C) because thermodynamic properties are easily obtained at this temperature in the open literature. In order to calculate the molar volume of the solvents at different temperatures and pressures, the Gunn-Yamada method [22] was used (Equation S5).

$$V(T) = \frac{f(T)}{f\left(T_{ref}\right)} \times V_{ref} \quad \text{(S5)}$$

In Equation S5, *f(T)* is a function of the selected temperature calculated using Equations S6(a-c).

$$f(T) = H_1(1 - \omega H_2) \quad \text{(S6-a)}$$

$$H_1 = 0.33593 - 0.33953T_r + 1.51941T_r^2 - 2.0251T_r^3 + 1.11422T_r^4 \quad \text{(S6-b)}$$

$$H_2 = 0.29607 - 0.090045T_r - 0.04842T_r^2 \quad \text{(S6-c)}$$

where, ω is the acentric factor and, T_r is the reduced temperature at the selected temperature.

When Equation S6(a) is solved, Equation S5 is easily calculated and can be substituted in Equations S4(a-c) to obtain the HSPs for the solvent. For the mixture, the HSP was obtained as the sum of the product between the volume fraction of EtOH or H_2O, and their corresponding HSP values when they were pure. Finally, HSPs for solute and solvent were substituted in Equation S1 to obtain the estimated *Ra* between molecules at high pressures and temperatures. After calculations, EtOH, EtOAc, and BnOH were selected as extraction solvents.

S1. Predictive step: Hansen solubility parameters (HSPs)

The first step was to estimate the HSPs for the solute. For that purpose, a Simplified Molecular Input Line Entry Specification (SMILES) was inserted in the HSPiP® software in "Do it Yourself" (DIY) tool, as seen in Figure S1. Using the Yamamoto molecular-break method, it was possible to obtain the contribution of each functional group to estimate the compound properties and the three HSP values. The HSPs found for piceatannol at 25 °C were: δ_D = 21.3, δ_P = 7.0, δ_H =10.4 and, δ_T = 24.7 MPa$^{1/2}$. The results implied a strong influence of the dispersion forces in the total solubility parameter, as this presented the highest value, which should be attributed to the two dihydroxybenzene groups in both piceatannol endings. Second, T_c of piceatannol was calculated. The groups used in T_c calculation for piceatannol are presented in Table S1. For the studied molecule, the presence of all order groups was taken into account.

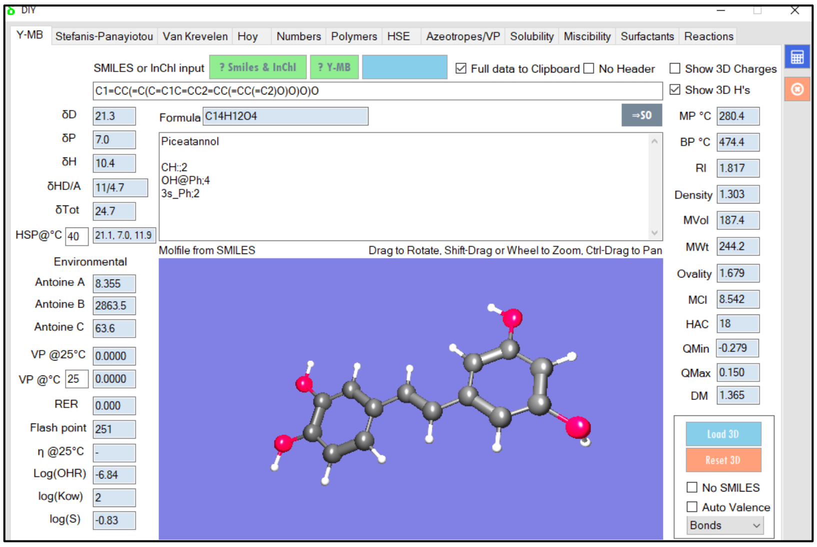

Figure S1. Interface of HSPiP® for HSPs calculation of Piceatannol using Yamamoto molecular breaker (Y-MB) group contribution method (at the standard condition: 25 °C and 0.1 MPa).

Table S1. Groups used in the Marrero and Gani group contribution method for the T_c determination of piceatannol.

**Piceatannol ($C_{14}H_{12}O_4$)**	Number of groups	T_c contribution (K) per group
First-order groups		
aCH	6	2.0337
aC-OH	4	9.3472
aC-CH	2	1.9512
Second-order groups		
AROMRINGs1s2	1	-0.3161
AROMRINGs1s3	1	-0.0693
Third-order groups		
aC-(CHn)m-aC (m>1)	1	3.0321

The T_c of piceatannol, obtained replacing the values presented in Table S1 in Equation S3, was 931.4 K (or 658.2 °C).

S2. Temperature influence on *Ra* estimative between solvents and piceatannol

Figure S2 presents de temperature dependence on *Ra* between the selected solvents and the target compound.

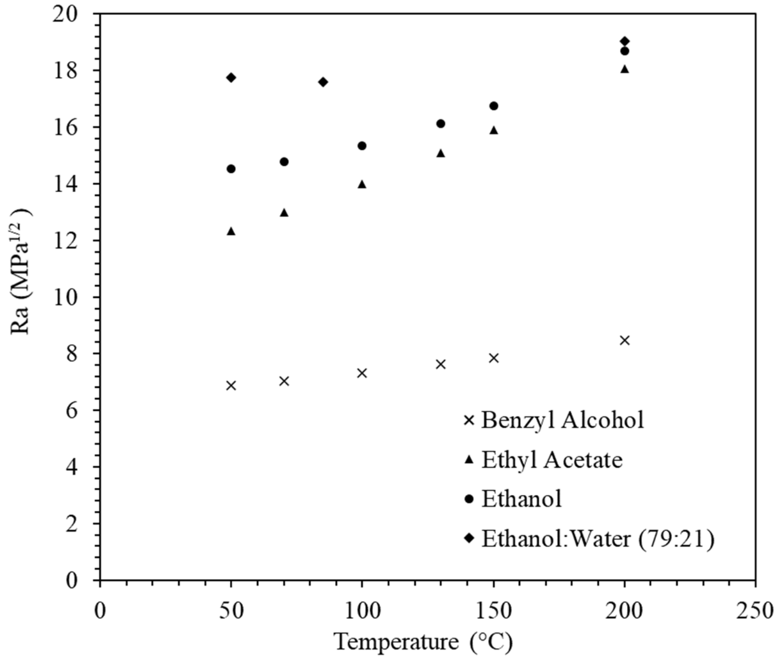

Figure S2. Temperature dependence of calculated *Ra* between bio-solvents and piceatannol.

The effect of temperature for all solvents (Benzyl alcohol – BnOH, Ethyl Acetate – EtOAc, Ethanol - EtOH) was negative for the target compound affinity once *Ra* values increased. The mixture of EtOH:H_2O applied in conventional extraction had the slightest increase of *Ra* in the evaluated temperature range (6.62%), followed by BnOH (18.79%), which is the solvent with lower *Ra* than the other solvents. EtOH and EtOAc presented the worst effects of temperature in values of *Ra*, with an increase in this parameter value (from 50 to 200 °C) of 22.34 and 31.60%, respectively. In this sense, Sánchez-Camargo et al. [38] also observed an elevated increase in *Ra* value for EtOAc (31.65%) when raising the process temperature to 100 °C, corroborating the estimates of this work. In practice, this rule is not applied, mainly because of the mass transfer kinetics. A multi-component system's complexity could directly affect the concentration of a specific compound class in the extract, as shown in this study for phenolics concentration and temperature process raising until 150 °C using EtOH as solvent.

S3. HSPs for pressurized BnOH

The molar volume of the liquids used as bio-solvents in this work were calculated as a function of temperature (Equation S5). The results for experimental density of BnOH at 25 °C and 0.1, 1, and 10 MPa were taken from Paknejad et al. [28]. Then, the molar volume was calculated and the HSPs are presented in Table S2.

Table S2. Influence of pressure on the HSPs of BnOH (108.14 g/mol) at 25 °C.

P (MPa)	V (cm^3/mol)	δ_D (MPa$^{1/2}$)	δ_P (MPa$^{1/2}$)	δ_H (MPa$^{1/2}$)	δ_T (MPa$^{1/2}$)
0.1	103.800	18.400	6.300	13.700	23.790
1	103.781	18.404	6.301	13.701	23.793
10	103.384	18.492	6.313	13.727	23.880

S4. PLE temperature effects

The color intensification of the extracts is presented in Figure S3.

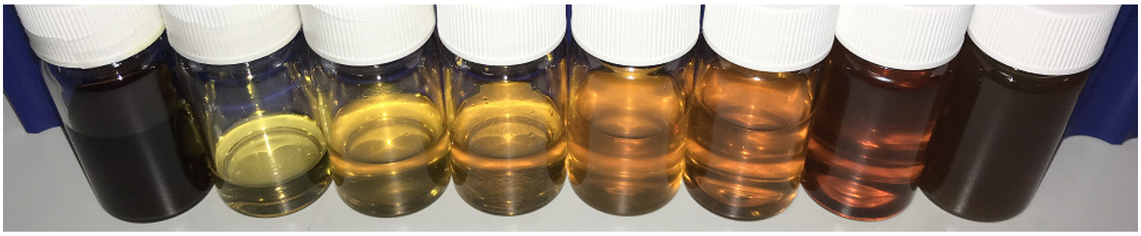

Figure S3. Temperature effect on PLE using EtOH as a solvent. Conventional extraction was performed using EtOH:H_2O (79:21) as solvent at 85 °C.

S5. Calibration curve of piceatannol

The standard calibration curve was built from standard dilutions in EtOH from 1-200 ppm every time a sample batch was run, and a representative curve is presented in Figure S4. The curves R^2 were: 0.9989, 0.9969, and 0.9991.

190.14x-128.95

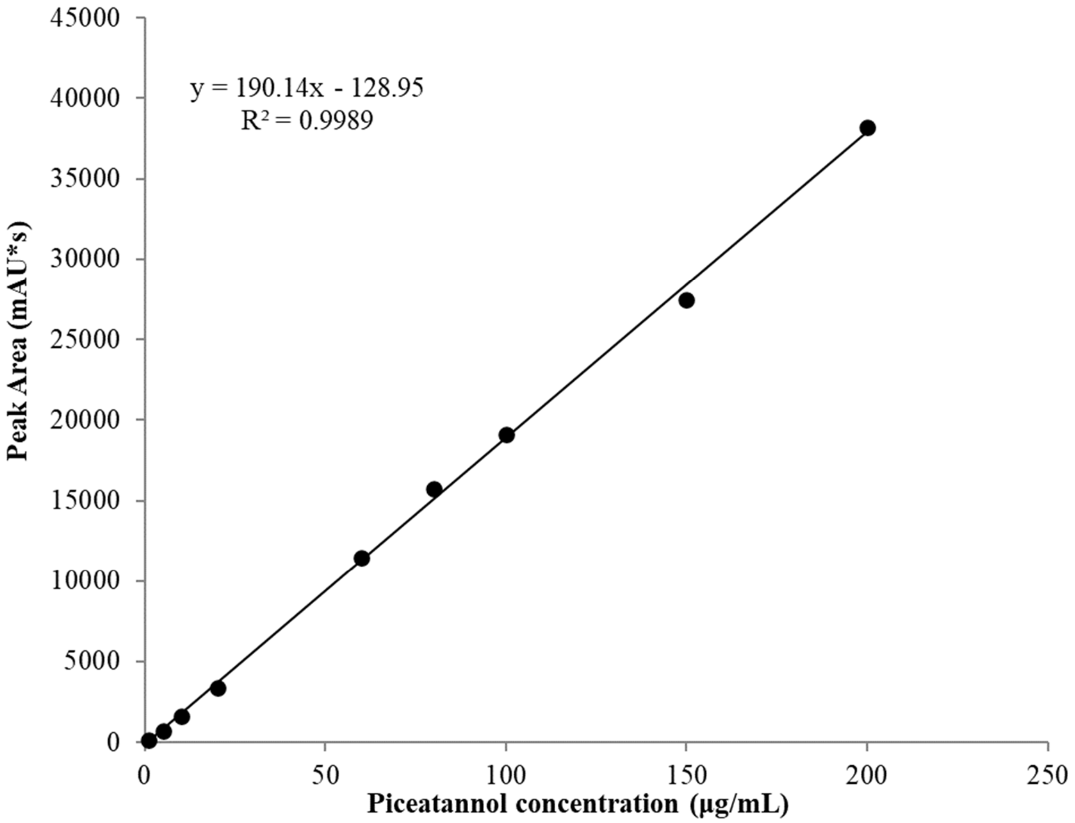

Figure S4. Calibration curve of piceatannol.

S6. Thermal stability test

After treating a 100 µg/mL solution of piceatannol in BnOH at different temperatures, they were analyzed by HPLC-DAD. Figure S5 shows the chromatograms obtained as well as the isoabsorbance plots in the range 260-700 nm and the graph homogeneity of the areas found using Fisher's least significant difference (LSD) procedure.

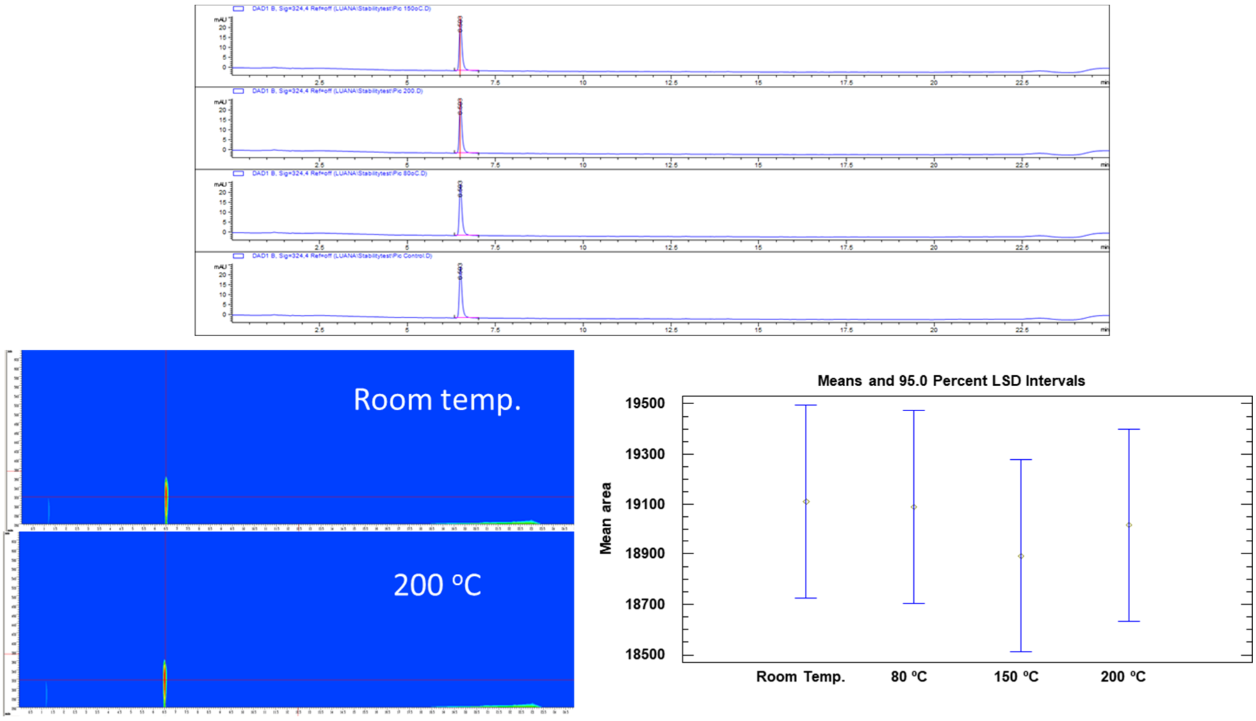

Figure S5. Chromatograms, isoabsorbance plots and area comparison of 100 µg/mL piceatannol in BnOH

S7. AChE Inhibitory effect of PLE EtOH 110 °C - cycle 1

The extract obtained by PLE with EtOH at 110 °C – cycle 1 did not reach 50% inhibition at the tested concentration range of 0.25-1.5 mg/ml (Figure S6). It was established a fixed concentration range for all extracts in order to have a fair comparison. Figure S6 confirms an increasing inhibitory behavior to extract concentration of approximately 100 µg/mL, achieving a maximum inhibition of 38.047 ± 1.359 µg/mL

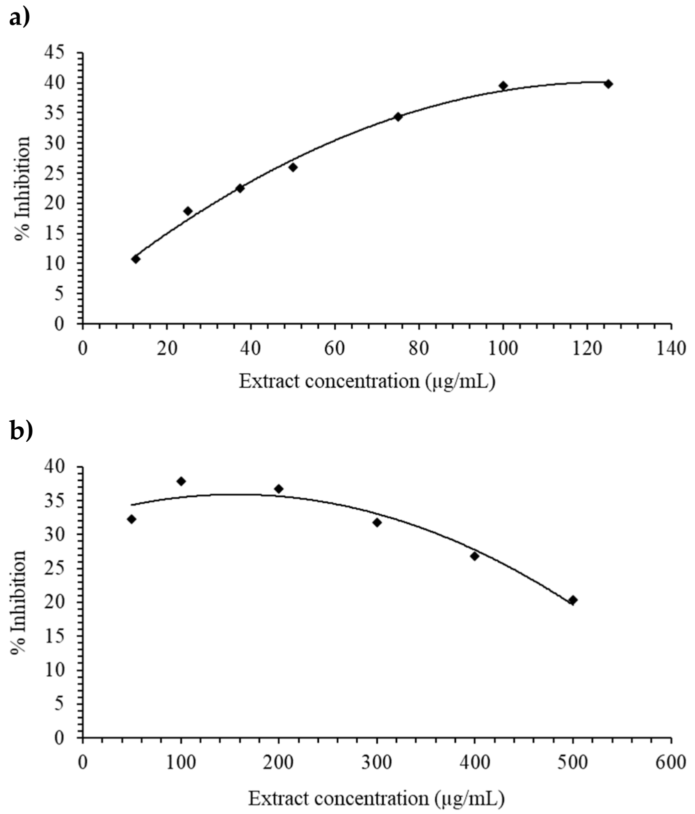

Figure S6. Enzymatic inhibition kinetics for acetylcholinesterase (AChE) using PFBP extract obtained with EtOH at 10 MPa and 110 °C (cycle 1). a) inhibition at extract concentration range of 12.5–125 µg/mL and, b) at 50 – 500 µg/mL.
